# Changes In Social Isolation, Anxiety and Depression Among Older Adults During The COVID-19 Pandemic

**DOI:** 10.1093/geroni/igab046.542

**Published:** 2021-12-17

**Authors:** Wenjun Li, Linda Churchill, Hannah Siden, Annabella Aquirre, Elizabeth Procter-Gray

**Affiliations:** University of Massachusetts Lowell, Lowell, Massachusetts, United States

## Abstract

Social distancing and business lockdowns may have severe negative impact on daily living, mental and physical health of community-living older adults. Our Healthy Aging and Neighborhood Study surveyed 370 older adults in Central Massachusetts in 2020 and 2021. Participants were queried about pre-post pandemic changes in social and physical activities, mental and physical health, and lifestyle factors including food purchasing, diet and physical exercise; and attitude towards and receiving of vaccination. The study is ongoing and data are being accumulated. Preliminary analysis suggested that social distancing and lockdowns have negative impacted social engagement, communications with close friends, relatives and family members, food purchasing, frequency of outdoor exercises, especially group activities. The impact appeared to differ by sex, advancing age, and living arrangement. In summary, social distancing and business lockdowns may have negative impacts on most older adults while the impacts were more severe in those older and socioeconomically disadvantaged.

